# Epidemiological, Clinical, and Biomarker Profile of Male Infertility in Morocco: A Retrospective Single-Center Study of 1399 Cases

**DOI:** 10.3390/diseases14010014

**Published:** 2025-12-30

**Authors:** Henri Hubert Kwizera Tsinda, Modou Mamoune Mbaye, Loïc Koumba, Reine Rolande Ada Edou, Achraf Zakaria, Noureddine Louanjli, Bouchra Ghazi, Fatima Maachi, Hakima Benomar, El Turk Joumana, Karima Sabounji

**Affiliations:** 1 Health Science Research Laboratory, Faculty of Health Sciences, International University of Casablanca, Casablanca 50169, Morocco; 2 Laboratory of Medical Analysis, Reproductive Biology, Labomac, Casablanca 20100, Morocco; 3 Immunopathology-Immunotherapy-Immunomonitoring Laboratory, Faculty of Medicine, Mohammed VI University of Health and Sciences, Casablanca 20270, Morocco; 4 IRIFIV Fertility Center, IVF Laboratory and MSREM Scientific Research Group, Casablanca 20250, Morocco; 5 Mohamed VI Center for Research and Innovation (CM6RI), Rabat 11103, Morocco

**Keywords:** male infertility, epidemiology, hormones, semen analysis, DNA damage, chromatin

## Abstract

Objective: The objectives of this study were to characterize the clinical, hormonal, and extended biomarker profile of infertile men in a Moroccan context, based on a retrospective single-center study, and to assess the relevance of selected markers for initial andrological assessment. Methods: This descriptive, retrospective, single-center study included 1399 men consulting for infertility between January and December 2024 in a specialized center. Collected data encompassed lifestyle habits, medical history, semen parameters (WHO 2021 criteria), sperm DNA fragmentation (TUNEL assay), nuclear decondensation, and hormonal assays (FSH, testosterone, and inhibin B) available in a subset of 156, 56, and 26 patients (for FSH, testosterone, and inhibin B, respectively). Associations with oligozoospermia were explored using univariate logistic regression analysis. Results: The mean age was 39.0 ± 8.0 years; 57% presented with primary infertility, and 82.8% were active smokers. A sperm concentration <16 M/mL was observed in 31.6% of patients. Among the 156 patients analyzed, high FSH levels were observed in 24% of cases. As for inhibin B, among the 26 patients evaluated, a decrease in levels was observed in 38% of cases. Pathological DNA fragmentation was found in 9.6%. In univariate analysis, oligozoospermia was significantly associated with elevated FSH (OR = 7.25; 95% CI: 3.15–16.70), varicocele (OR = 1.81), and smoking (OR = 0.66). Conclusion: This is the first large-scale Moroccan study integrating advanced biomarkers into the assessment of male infertility. The observed associations between elevated FSH, sperm DNA fragmentation, and varicocele support the development of a simplified andrological triage strategy, particularly relevant in resource-limited settings.

## 1. Introduction

Male infertility accounts for nearly 50% of infertility cases in couples, either as an isolated cause or in combination with a female factor [1]. Despite its substantial epidemiological burden, high-quality data describing the clinical, biochemical, and hormonal characteristics of male infertility remain unevenly distributed worldwide. In particular, low- and middle-income countries are underrepresented in international scientific literature, notably in North and sub-Saharan Africa [2]. This paucity of data limits the understanding of regional specificities, hinders the development of tailored preventive strategies, and delays the integration of efficient diagnostic approaches into male reproductive health care pathways.

In resource-limited settings, the evaluation of infertile men still largely relies on conventional semen analysis, despite its well-established limitations in predictive value. In recent years, several biomarkers have been proposed to refine andrological assessment, including follicle-stimulating hormone (FSH, reflecting the gonadotroph axis), inhibin B (a marker of Sertoli cell function), and sperm DNA integrity (assessed by assays such as TUNEL).

Specifically, follicle-stimulating hormone (FSH) is a key indicator of the pituitary–gonadal axis; its elevation often reflects a lack of negative feedback from the tests, typically signaling impaired spermatogenesis and reduced testicular secretory function. Inhibin B, a dimeric peptide secreted by the Sertoli cells, serves as a more direct marker of Sertoli cell function and, consequently, of exocrine testicular activity, with low levels being highly indicative of severe spermatogenic impairment. Furthermore, assessing sperm DNA integrity, for instance via the TUNEL assay, is critical as high levels of DNA fragmentation are associated with reduced natural conception potential and lower success rates in Assisted Reproductive Technologies (ART), independent of conventional semen parameters.

These markers have demonstrated clinical utility for stratifying the risk of testicular dysfunction, predicting outcomes in assisted reproductive technologies (ART), and tailoring management strategies [3,4].

However, no consensus andrological triage strategy has yet been validated in countries of the Global South, one that would integrate basic semen parameters with hormonal data and chromatin or behavioral markers. The absence of a simplified clinical algorithm incorporating these variables limits the early referral of patients to specialized testing or ART, representing a missed opportunity, particularly in fragmented health care systems.

In Morocco, the limited available data derive from hospital-based, retrospective studies, often restricted to semen analysis or basic hormonal profiling. Few investigations have incorporated advanced biomarkers such as sperm DNA fragmentation or inhibin B, nor have they jointly analyzed lifestyle factors, clinical history, and paraclinical data within a triage-oriented framework.

Against this backdrop, our study seeks to address both the geographic and methodological gaps by characterizing the sociodemographic, clinical, hormonal, and chromatin profiles of a large cohort of infertile men managed in a private specialized center in Morocco. It also examines the discriminative value of selected biomarkers in predicting severe spermatogenic impairment.

Our working hypothesis is as follows: a limited panel of accessible markers (FSH, varicocele, DNA fragmentation) could significantly improve the initial triage of infertile patients, particularly in resource-limited settings where specialized tests are not always readily available.

## 2. Materials and Methods

### 2.1. Study Population

This retrospective descriptive study included all male patients consulting for infertility at the Labomac Medical Biology Laboratory in Casablanca between January and December 2024, totaling 1431 records. Informed consent was obtained prior to any inclusion, in accordance with the ethical principles of the Declaration of Helsinki.

### 2.2. Inclusion Criteria

Patients were required to have a documented history of infertility, defined as the absence of conception after at least 12 months of regular unprotected sexual intercourse.

### 2.3. Exclusion Criteria

The exclusion criteria were designed to eliminate any major source of clinical or biological bias. All incomplete records or those lacking results from semen analysis, hormonal profiling, or standardized clinical history-taking were excluded from the analysis.

### 2.4. Data Collection

Data were collected using a structured survey form completed by the biologist or andrologist, comprising predefined fields covering sociodemographic, medical, clinical, and biological parameters. The data was extracted from medical records archived at the laboratory. The study was conducted in compliance with confidentiality requirements and in accordance with Moroccan legislation on the protection of personal data.

### 2.5. Sociodemographic Data and Lifestyle Habits

Sociodemographic variables included chronological age (expressed in years) and body mass index (BMI, kg/m^2^), calculated from reported anthropometric measurements. Occupational status was coded according to the level of sedentary activity and degree of exposure to thermal or toxic agents known to potentially affect male fertility. Lifestyle habits included the use of tobacco, alcohol, and cannabis, as well as engagement in regular physical activity. These data were obtained through a structured interview during an andrological consultation, with the aim of identifying behavioral exposures potentially impacting testicular function.

### 2.6. Clinical Data and Medical History

Clinical variables included the type of infertility (primary or secondary) and the duration of infertility, expressed in years since the onset of unprotected conception attempts. The medical history assessment targeted factors likely to impair testicular function, including the presence of varicocele, cryptorchidism, prior herniorrhaphy, or a history of testicular torsion in surgical records. Infectious history included documented upper urogenital infections and self-reported sexually transmitted infections (STIs). COVID-19 vaccination status (at least one dose) and the presence of known type 2 diabetes were systematically recorded. In addition, patients were questioned regarding the use of potentially gonadotoxic medications (exogenous androgens, chemotherapeutic agents, anticonvulsants) as well as the presence of first-degree family history of male infertility.

### 2.7. Biological and Paraclinical Data

Semen analysis was performed in accordance with the recommendations of the 6th edition of the World Health Organization (WHO) Laboratory Manual for the Examination and Processing of Human Semen (2021), applying the following reference thresholds: ejaculate volume ≥ 1.4 mL, sperm concentration ≥ 16 million/mL, progressive motility (grades a + b) ≥ 32%, vitality (eosin–nigrosine stain) ≥ 58%, and normal morphology ≥ 4%, assessed according to Kruger’s strict criteria. Physicochemical parameters also included pH ≥ 7.2 and normal viscosity, defined as a thread length < 2 cm.

Sperm chromatin quality was assessed in a subset of 385 patients. DNA fragmentation was evaluated using the TUNEL (Terminal deoxynucleotidyl transferase dUTP nick end labeling) assay with the In Situ Cell Death Detection Kit, Fluorescein^®^ (Roche Diagnostics GmbH, Mannheim, Germany). Smears were prepared from methanol-fixed sperm cytospins, followed by enzymatic digestion with proteinase K, incubation with terminal deoxynucleotidyl transferase (TdT) enzyme, and labeling with fluorescein-conjugated dUTP. Analysis was performed using fluorescence microscopy (FITC filter), with manual counting of 200 spermatozoa per sample. A positivity threshold of ≥30% fluorescent spermatozoa was applied to define pathological fragmentation, in accordance with published standards [5].

Nuclear decondensation was assessed in parallel, when feasible, either by aniline blue staining or by chromomycin A3 (CMA3) testing. A rate of ≥30% abnormal cells was considered pathological, indicating altered chromatin compaction.

Hormonal assays were performed in an accredited medical biology laboratory using the IMMULITE^®^ 2000 analyzer (Siemens Healthineers, Tarrytown, USA). The reference ranges applied were as follows: FSH (1.5–12.4 mIU/mL), total testosterone (2.7–10 ng/mL), and inhibin B (80–400 pg/mL). Pathological thresholds were defined as FSH > 12.4 mIU/mL, inhibin B < 80 pg/mL, and total testosterone < 2.7 ng/mL, reflecting, respectively, impaired testicular secretory function, reduced exocrine spermatogenesis, and androgen axis deficiency.

### 2.8. Statistical Analysis

Data were analyzed using SPSS software, version 25 (IBM Corp., Armonk, NY, USA). Quantitative variables are expressed as means ± standard deviation, and qualitative variables as counts and percentages. Statistical tests, including the chi-square test, Student’s *t*-test, and Spearman’s correlation or logistic regression analyses, were employed to assess associations between clinical, biological, and sociodemographic parameters.

### 2.9. Ethical Considerations

The protocol was approved by the Biomedical Research Ethics Committee of UM6SS (reference: CE/UM6SS/09/23). All data were analyzed anonymously in accordance with the principles of the Declaration of Helsinki.

## 3. Results

### 3.1. Composition and Selection of the Analytical Cohort

A rigorous selection process was applied. Inclusion criteria required documented infertility, defined as the absence of conception after ≥12 months of regular unprotected intercourse. Exclusion criteria included: (i) incomplete records, (ii) absence of semen analysis, (iii) absence of a hormonal profile or standardized medical history, and (iv) insufficient clinical data for analysis.

**Figure 1:**
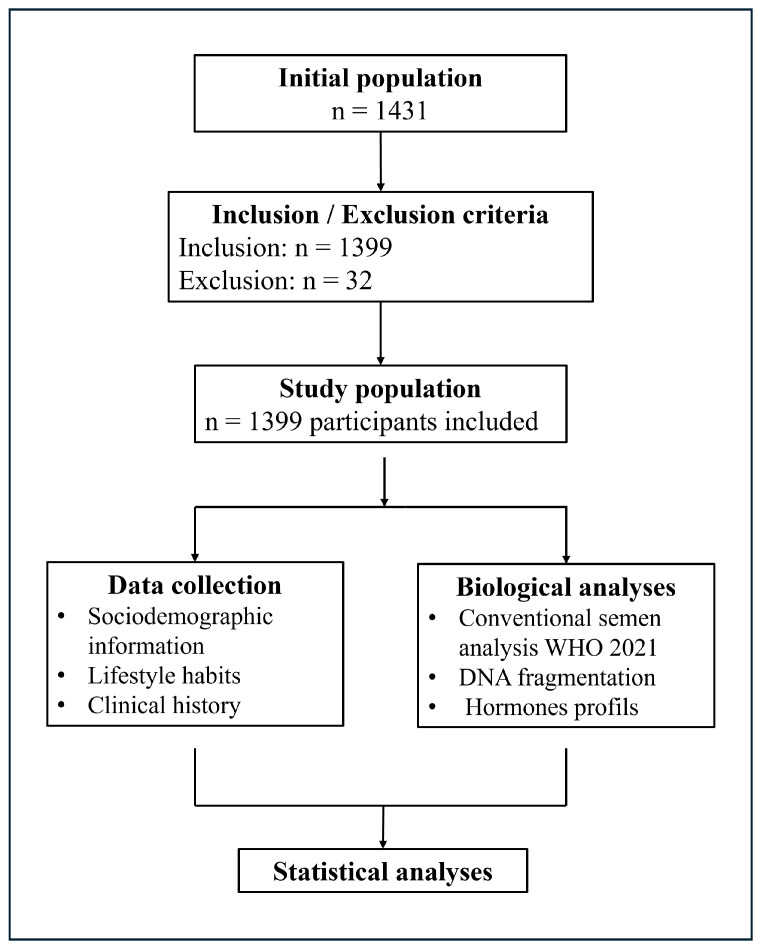
Flow diagram of the retrospective study: patient selection and analysis methodology.

In total, 32 records were excluded, resulting in a final cohort of 1399 patients included in the retrospective analysis (Figure 1).

This selection process ensures data homogeneity and enhances the reliability of subsequent analyses.

### 3.2. Distribution of Ages and Sociodemographic Characteristics of Patients

**Figure 2:**
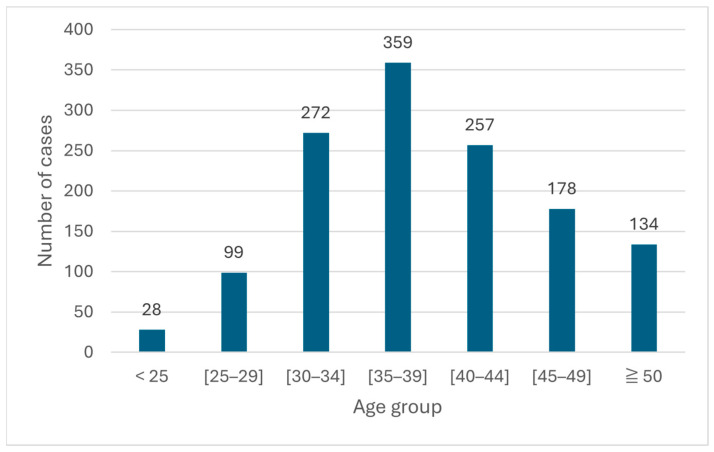
Distribution of patients by age group.

The mean age of patients was 39.0 ± 8.0 years (range: 16–66 years), indicating that most andrology consultations occurred at an age still considered optimal for male reproduction. Age-group distribution showed a predominance of patients aged 35–39 years (26.0%) and 40–44 years (21.0%), corresponding to the peak of male reproductive demand. In contrast, men under 25 years and those aged 50 years and older accounted for a small proportion of consultations (2.0% and 10.0%, respectively), suggesting a potential underutilization of reproductive health services in these age groups (Figure 2).

### 3.3. Type and Duration of Infertility: Consultation Profile and Diagnostic Delays

In this cohort, primary infertility was the predominant form, accounting for 57.0% of cases (*n* = 721), compared with 43.0% for secondary infertility (*n* = 554).

**Figure 3:**
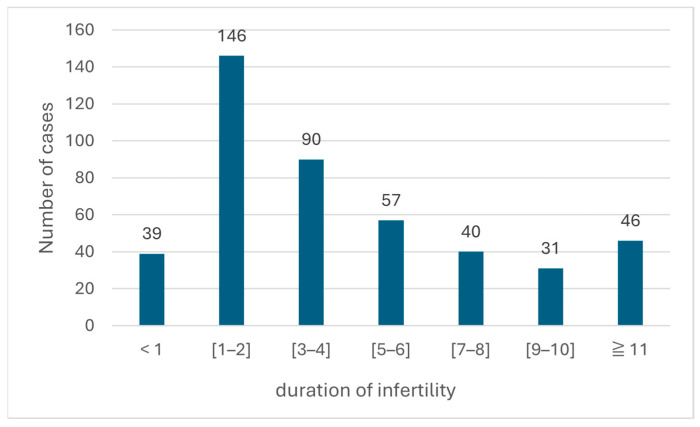
Distribution of patients according to duration of infertility.

Analysis of infertility duration (Figure 3) revealed considerable variability in the delay to seek medical evaluation. More than half of the patients (61.2%) sought medical consultation within the first five years after attempting conception, with a marked peak between 1 and 2 years (n = 146; 32.5%). However, approximately one-third of men (26.1%) had experienced infertility for more than seven years, and nearly 10% (n = 46) waited over 11 years before undergoing specialized assessment.

### 3.4. Lifestyle Factors, Comorbidities, and Spermiological Profile: Lifestyle Factors, Comorbidities, and Semen Parameters

The reported lifestyle habits in the cohort revealed a very high prevalence of smoking, with 82.8% of men identifying as active smokers (*n* = 1158) compared with only 17.2% non-smokers. In contrast, regular alcohol consumption was marginal (5.8%), possibly reflecting cultural habits in this population. Occupational data were available for only a small subsample (*n* = 62; 4.4%), of whom 62.9% were engaged in highly sedentary work, a condition that may predispose to scrotal hyperthermia; the limited sample size precludes firm conclusions. Clinically, varicocele was present in 9.4% of patients (by clinical examination), with 3.5% presenting in an isolated form, underscoring the importance of systematic screening during the initial evaluation. Furthermore, 5.3% of men reported the use of potentially gonadotoxic medications (androgens, chemotherapy, anticonvulsants), and 3.0% had diabetes, although this latter factor was rarely isolated (0.5% of cases with no overlapping lifestyle factors), making it difficult to assess diabetes as an isolated determinant.

**Figure 4:**
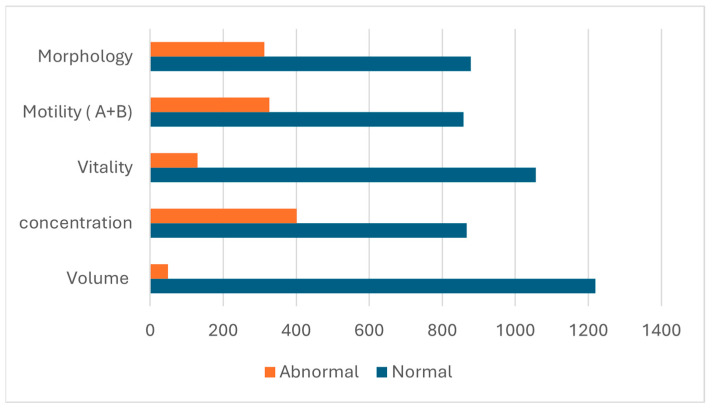
Distribution of patients according to semen profile.

**Figure 5:**
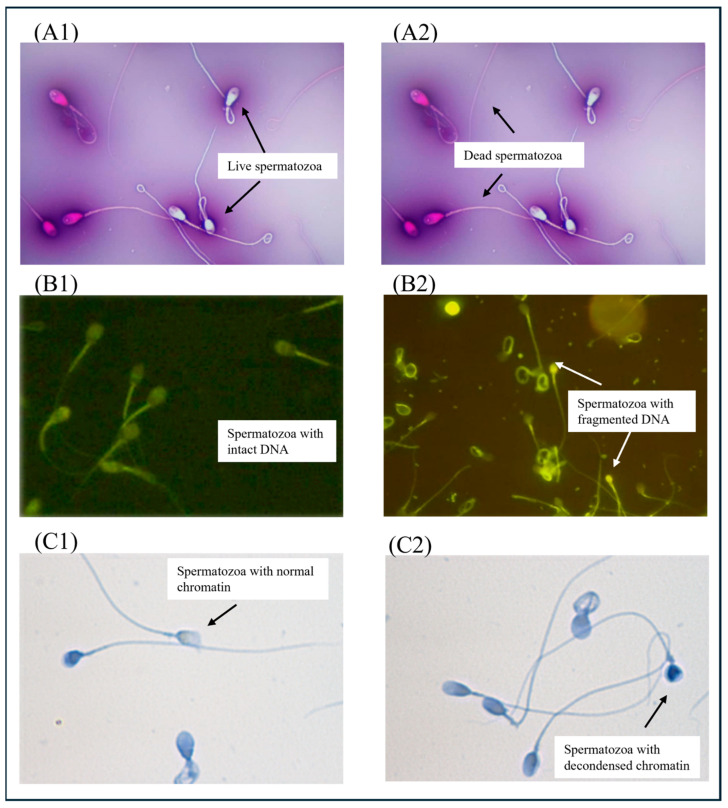
Microscopic images illustrate the assessment of sperm biological quality. (**A**) Assessment of vitality by eosin-nigrosine staining, showing live (unstained **A1**) and dead (stained **A2**) spermatozoa. (**B**) Assessment of DNA fragmentation using the TUNEL assay, showing fragmented DNA (green fluorescent nuclei, arrow **B2**) and intact DNA (non-fluorescent nuclei **B1**). (**C**) Assessment of nuclear decondensation using aniline blue staining, showing abnormal chromatin compaction (stained nuclei, arrow **C2**) and normal compaction (unstained nuclei **C1**).

Biologically, semen analysis performed according to WHO 2021 standards demonstrated good compliance with pre-analytical conditions (3–5 days of abstinence in 89.7% of cases) and normal ejaculate volume in 96.1% of cases [6]. However, functional parameter alterations were frequent: only 68.4% of patients exhibited a normal sperm concentration (≥16 M/mL), and 72.5% reached the threshold for progressive motility (≥32%), indicating compromised kinetics in nearly one-third of cases. Morphological assessment, performed according to Kruger’s strict criteria, revealed that 73.7% of patients had a normal morphology rate of ≥4%, suggesting overall preservation of morphogenesis. Nevertheless, nearly one in four patients presented with diagnostic teratozoospermia, warranting a combined interpretation of kinetic, morphological, and chromatin parameters (Figure 4). Cell vitality remained preserved (≥58%) in 89.0% of cases, reflecting overall viability despite these abnormalities (the staining image is shown in Figure 5).

### 3.5. Chromatin Quality and Hormonal Profiles: Complementary Exploration of Testicular Alterations

Sperm chromatin quality was assessed in a subset of 385 patients using two complementary tests: the DNA fragmentation index (TUNEL assay) and nuclear decondensation analysis (aniline blue staining); the staining images are shown in Figure 5. The majority of profiles showed good chromatin integrity. Indeed, 90.4% of patients exhibited a DNA fragmentation rate below the pathological threshold of 30%, whereas 9.6% exceeded this cut-off, indicating potential impairment of sperm genetic material. Similarly, nuclear decondensation was within normal limits in 90.1% of individuals and pathological in 9.9%.

In parallel, a hormonal evaluation was performed in a second subset of patients, with sample sizes varying according to the assay: 156 patients for FSH, 56 for total testosterone, and only 26 for inhibin B. FSH, the principal marker of testicular secretory function, was within the normal range in 75.6% of cases but elevated in 23.7%, indicating possible spermatogenic impairment. Total testosterone levels were within physiological ranges in 82.1% of subjects, while biochemical androgen deficiency was suspected in 14.3%. Finally, inhibin B, although assessed in a very limited number of patients, was reduced in 38.5%, reflecting a more specific impairment of Sertoli cell function and exocrine spermatogenesis (Table 1).

### 3.6. Comparability of the Hormonal Subgroup: Methodological Validation of the Analyzed Subsample

To ensure the validity of endocrine analyses performed on a restricted subgroup, a methodological comparison was conducted between the 156 patients who underwent hormonal testing and the remaining 1243 individuals (Table 2). No significant differences were observed in the main semen parameters, including sperm concentration (*p* = 0.060), progressive motility (*p* = 0.051), and morphology according to Kruger (*p* = 0.391), suggesting biological homogeneity between the two groups. Similarly, the distribution of infertility types (primary vs. secondary), the prevalence of varicocele, and smoking status did not differ significantly. Only mean age showed a moderate but statistically significant variation (37.6 ± 6.9 years in the hormonal group vs. 39.7 ± 8.0 years; *p* = 0.002), without compromising the overall representativeness of the subsample.

### 3.7. Comparison of Semen Profiles Between Primary and Secondary Infertility: Age and Semen Parameters

Comparative analysis of semen characteristics between patients with primary infertility (*n* = 338) and those with secondary infertility (*n* = 55) revealed a significant age difference, with the latter being, on average, older (45.5 ± 8.46 years vs. 39.0 ± 7.34; *p* < 0.001). Conversely, no conventional semen parameter (concentration, motility, morphology, or duration of infertility) significantly differentiated the two groups (Table 3).

### 3.8. Impact of Varicocele on Semen Parameters: Significant Quantitative and Kinetic Impairment

Comparative analysis of semen profiles between patients with varicocele (*n* = 124) and those without varicocele (*n* = 589) revealed several significant differences. Men with varicocele were, on average, younger (36.0 ± 8.29 years) than those without (39.5 ± 8.14 years; *p* < 0.001), likely reflecting earlier diagnosis in symptomatic cases. Qualitatively, progressive motility was significantly reduced in affected patients (37.3 ± 19.1% vs. 42.3 ± 17.4%; *p* = 0.0072), as was sperm concentration (44.2 ± 43.2 M/mL vs. 60.0 ± 50.0 M/mL; *p* < 0.001), indicating both functional and quantitative impairment of spermatogenesis. In contrast, morphology and vitality did not differ significantly between groups (Table 4).

### 3.9. Factors Associated with Oligozoospermia: Discriminative Contribution of FSH and Varicocele in Univariate Analysis

Univariate analysis of clinical and biological determinants of oligozoospermia (sperm concentration < 16 M/mL) identified several significant associations (Table 5). Elevated FSH emerged as the strongest predictive factor, with a crude odds ratio of 7.25 (95% CI: 3.15–16.70; *p* < 0.001), reflecting the high diagnostic value of this marker in testicular secretory dysfunction. The presence of varicocele was also significantly associated with oligozoospermia (OR = 1.81; 95% CI: 1.23–2.67; *p* = 0.003), confirming its detrimental effect on spermatogenesis. In contrast, the association with smoking was inverse (OR = 0.66; *p* = 0.046), suggesting a potential selection bias or a confounding effect related to other environmental factors. Age was not significantly correlated with sperm concentration. Secondary hormonal markers, such as inhibin B and testosterone, could not be included in the models due to unstable results or lack of statistical convergence, related to the small size of the subsamples.

## 4. Discussion

The present study, conducted on a large cohort of 1399 infertile men, represents one of the few comprehensive analyses that simultaneously integrates sociodemographic, clinical, hormonal and chromatin-related biomarkers within a North African context. In contrast with previous studies from Morocco, which were often restricted to the evaluation of conventional semen parameters, our investigation adopts an integrated andrological profiling approach. It applies to the revised WHO 2021 criteria with strict methodological rigor and includes extended indicators such as sperm DNA fragmentation and serum inhibin B. This methodological framework gives our findings substantial translational relevance, particularly for guiding clinical triage strategies in settings where medical resources are limited.

### 4.1. Epidemiological Profile: Advanced Age and Predominance of Primary Infertility

The mean age of 39 years observed in our population is consistent with findings from Mediterranean and Middle Eastern cohorts, where delayed andrological consultation is frequently reported [7,8]. This trend may reflect a lack of awareness regarding male fertility, as well as sociocultural patterns in which men seek medical attention only after several years of undocumented infertility, often based on the initial assumption of a female-related cause.

The high proportion of 57% of primary infertility supports global data indicating that men typically undergo a complete andrological evaluation only after an extended period of unsuccessful attempts to conceive, often exceeding the twelve-month timeframe recommended for clinical assessment [2]. This delayed consultation is particularly concerning given that advanced paternal age is now recognized as a significant reproductive factor.

Indeed, several recent studies have shown that increasing male age is associated with higher oxidative stress, elevated sperm DNA fragmentation, and epigenetic alterations. These factors negatively impact not only fertility potential but also offspring health [8,9,10]. These observations highlight the importance of early screening in regions where delayed consultation remains a sociocultural norm.

### 4.2. Lifestyle Factors: Exceptionally High Prevalence of Smoking

In our cohort, the prevalence of smoking reached 82.8%, a level exceptionally high compared to previously published series. This finding is particularly concerning in light of recent evidence. A major meta-analysis by Sharma et al., involving 5865 men, demonstrated that smoking is significantly associated with reduced sperm concentration (−9.7 × 10^6^/mL), progressive motility (−3.5%), and normal morphology [11]. Similarly, the meta-analysis by Bundhun et al. confirmed that smokers exhibit a general decline in semen quality, including reduced concentration, total motile sperm count, and normal morphology [12]. More recently, Ramon et al., in a meta-analysis of over 12,000 men, showed that smoking is not only associated with altered semen parameters, but also with hormonal disturbances involving FSH, LH, and testosterone, suggesting a combined impact on the hypothalamic–pituitary–gonadal axis and spermatogenesis [13].

From a pathophysiological perspective, chronic exposure to nicotine, polycyclic aromatic hydrocarbons, and heavy metals induces significant oxidative stress, impairs mitochondrial function, alters the expression of stress proteins such as HSP27, and disrupts testicular vascularization [14,15]. These mechanisms explain the increase in sperm DNA fragmentation, the alteration of membrane fluidity, and the decline in motility observed in smokers.

The extraordinarily high prevalence of smoking in our population may therefore represent a major determinant of the sperm abnormalities identified. This fully supports the systematic integration of smoking cessation into male infertility management strategies, especially in resource-limited settings.

Occupational sedentariness, reported by 62.9% of respondents (albeit in a small subsample), may also represent an indirect contributing factor, potentially related to prolonged exposure to pelvic heat or low levels of physical activity. This is consistent with recent studies showing that a sedentary lifestyle promotes hypogonadism and deteriorates semen quality in men, and that physical inactivity is associated with an increased risk of male infertility [16].

### 4.3. Semen Parameters: Compromised Motility and Interindividual Variability

Although the majority of patients presented with normal semen volume and vitality, nearly one third did not reach the threshold for progressive motility (≥32%). This kinetic impairment is comparable to that observed in large European and Asian studies [1,17], and may result from multiple factors including oxidative stress, varicocele, smoking, chronic thermal exposure, or genital inflammation. The strict application of the WHO 2021 criteria reinforces the reliability of our interpretation, as these standards are recognized for their precision and improved clinical predictive value. Regarding morphology, the proportion of normal forms, observed in 73.7% of cases according to Kruger’s strict criteria, appears encouraging but must be interpreted with caution. Morphological abnormalities are frequently underestimated in non-specialized laboratories [17].

### 4.4. Chromatin Biomarkers: DNA Fragmentation and Chromatin Decondensation

In our cohort, 9.6% of men exhibited elevated DNA fragmentation (TUNEL ≥ 30%). This relatively low rate should be interpreted with caution due to: (i) the selective nature of the test indication, (ii) the inter-laboratory variability inherent to the TUNEL technique (iii) the high threshold applied (≥30%), while recent studies suggest that a cutoff of 20–25% may better predict ICSI failure or miscarriage [18,19,20,21].

It is widely established that sperm DNA fragmentation is associated with reduced pregnancy rates and increased early pregnancy loss [5,22]. Recent studies also confirm that it may inform therapeutic strategies, particularly the use of testicular spermatozoa in cases of repeated implantation failure [23,24].

### 4.5. Hormonal Profile: Central Role of FSH and Clinical Relevance of Inhibin B

In our cohort, elevated FSH levels were observed in 23.7% of cases, confirming the central role of this marker as an indicator of testicular dysfunction and exocrine hypogonadism. These findings are consistent with literature data showing that FSH is one of the most sensitive hormonal biomarkers for detecting impaired spermatogenesis, particularly in cases of severe oligozoospermia or non-obstructive azoospermia [25].

Inhibin B, although measured in a smaller subset of patients in our study, was decreased in 38.5% of cases. This reduction represents a strong biological signal reflecting significant impairment of Sertoli cell function and, consequently, spermatogenesis. Recent studies support this interpretation, showing that inhibin B is among the best predictive markers of spermatogenic efficiency and sperm retrieval potential in azoospermic men [26]. Similarly, Deng et al. demonstrated that inhibin B, especially when combined with other markers such as FSH or integrated into hormonal ratios, significantly improves diagnostic accuracy for predicting sperm retrieval success during testicular sperm extraction (TESE), particularly in non-obstructive azoospermia [27].

Thus, despite the limited number of hormonal assessments in our cohort, our results align with a strong pathophysiological rationale: the combination of high FSH and low inhibin B remains one of the endocrine profiles most strongly correlated with severe spermatogenic failure and represents a key triage tool to guide patients toward appropriate diagnostic or therapeutic interventions.

The limited availability of hormonal assays in peripheral centers in Morocco gives these results particular clinical importance in the context of patient triage.

### 4.6. Varicocele: Confirmed Strong Negative Impact

In our cohort, varicocele was identified in 9.4% of infertile men. This prevalence aligns with international trends and highlights the importance of systematic screening for varicocele during andrological consultations. Our results show that men with varicocele presented significant alterations in semen parameters compared to non-affected individuals. Sperm concentration was markedly lower in patients with varicocele (44.2 ± 43.2 M/mL) compared to those without (60.0 ± 50.0 M/mL; *p* < 0.001). Similarly, progressive motility was significantly reduced (37.3 ± 19.1% vs. 42.3 ± 17.4%; *p* = 0.007), while morphological parameters and vitality showed no significant differences. These selective alterations, primarily affecting concentration and motility, are consistent with classical varicocele-related mechanisms such as scrotal hyperthermia, testicular hypoxia, venous stasis, and elevated oxidative stress.

Recent studies by Nakonechnyi et al. and Lira Neto et al. confirm that varicocele is strongly associated with spermatogenic impairment, increased DNA fragmentation, and mitochondrial dysfunction that directly impacts motility. A notable finding in our study is that men with varicocele were significantly younger (36.0 ± 8.29 years vs. 39.5 ± 8.14 years; *p* < 0.001), which may reflect earlier detection of sperm abnormalities in this subgroup [28,29].

These observations reinforce the relevance of routine scrotal examination during the initial infertility evaluation and support the role of varicocelectomy as a therapeutic option likely to improve both quantitative and functional sperm parameters.

### 4.7. Univariate Analysis: FSH as a Discriminant Biomarker

Our univariate analysis identified elevated follicle-stimulating hormone (FSH) as the variable most strongly associated with oligozoospermia, with an odds ratio (OR) of 7.25, a value within the upper range of international estimates. This finding supports the relevance of FSH as a discriminant hormonal biomarker, allowing for the early identification of patients with severely impaired spermatogenesis. The literature highlights that elevated FSH reflects a reduction in the inhibitory feedback exerted by Sertoli cells, which is itself linked to fewer functional seminiferous tubules.

Moreover, the significant association between varicocele and oligozoospermia observed in our cohort reinforces the importance of considering this diagnosis as a priority therapeutic target, particularly in contexts where surgical treatment may improve sperm concentration and motility. This result is consistent with meta-analyses showing that varicocelectomy improves semen parameters and reduces sperm DNA fragmentation [30,31].

Finally, the inverse association between smoking and oligozoospermia observed in our analysis must be interpreted with caution. This result contradicts the existing literature, where smoking is clearly associated with decreased sperm concentration and motility, and may reflect selection bias, possibly related to screening habits, socioeconomic profiles, or underreporting of tobacco use among more severely affected patients. Further adjusted multivariate analyses or prospective studies are needed to clarify this paradoxical finding.

### 4.8. Originality and Contribution of the Study

This study is distinguished by several elements that enhance its scientific and clinical relevance in the field of male infertility in Morocco. It represents the first large Moroccan cohort to simultaneously include hormonal profiling (FSH, testosterone, inhibin B), sperm DNA fragmentation analysis, nuclear decondensation evaluation, and rigorous application of the updated WHO 2021 criteria, thereby offering a multidimensional approach rarely documented in the region.

The large and homogeneous sample size (*n* = 1399) provides robust analytical power, allowing precise exploration of interindividual variation in reproductive parameters. In addition, our study includes relevant subgroup analyses, particularly according to type of infertility (primary or secondary), age, or the presence of varicocele, which significantly enrich the pathophysiological interpretation of the results.

The study’s originality also lies in its clinical triage orientation, specifically designed for resource-limited settings, where access to advanced biomarkers remains restricted.

Finally, when compared to the recent study by Rochdi et al., which involved a larger cohort but focused mainly on sociodemographic and occupational factors, our work goes further by integrating structural and functional sperm biomarkers, thereby strengthening its value in guiding diagnostic and therapeutic strategies in andrology [32].

### 4.9. Limitations and Perspectives

This study presents several limitations related to its design and the availability of data. First, its retrospective nature exposes it to selection bias and to heterogeneity in the information recorded in medical files. Hormonal assays and inhibin B measurements were only performed in a limited subset of patients, which restricts the scope of endocrine conclusions and prevents the construction of robust multivariate models. Likewise, the absence of longitudinal data on clinical outcomes such as pregnancy rates, miscarriage rates, or IVF/ICSI performance limits the ability to assess the true predictive value of the studied biomarkers. In addition, the analysis of advanced kinetic parameters through CASA, such as VCL, VAP, and ALH, which would have allowed for a more refined understanding of sperm dynamics, could not be systematically integrated due to limitations inherent in retrospective data collection. Considering these considerations, prospective and ideally multicenter studies are needed, with standardized collection of hormonal and chromatin biomarkers, as well as follow-up of reproductive outcomes, to validate a national clinical triage algorithm. Such an algorithm could optimally incorporate simple but informative parameters such as FSH, inhibin B, presence of varicocele, DNA fragmentation rate, smoking status, and urogenital history, with the aim of improving the management of infertile men in resource-limited settings.

## 5. Conclusions

This study provides a comprehensive and representative overview of the sociodemographic, clinical, and semen characteristics of infertile men consulting in a specialized reproductive health center in Morocco. The high prevalence of smoking, predominance of primary infertility, and frequent motility impairments reflect key modifiable and biological factors contributing to male infertility in this setting. Although hormonal analyses were available only for a subset of patients, the observed elevations in FSH and reductions in inhibin B suggest impaired exocrine testicular function. These findings call for the systematic inclusion of hormonal profiling in initial infertility assessments and support the implementation of early, multidimensional screening strategies to optimize male reproductive health care in resource-limited contexts.

## Figures and Tables

**Table 1:** Distribution of hormonal profile according to the standard reference range.

Hormonal Parameter	Total (*n*)	Normal *n* (%)	Low *n* (%)	High *n* (%)
FSH	156	118 (75.6%)	1 (0.6%)	37 (23.7%)
Testosterone	56	46 (82.1%)	8 (14.3%)	2 (3.6%)
Inhibin B	26	16 (61.5%)	10 (38.5%)	0 (0%)

**Table 2:** Comparison of clinical and biological characteristics between patients with and without hormonal assessment.

Variable	With Hormonal Test (*n* = 156)	No hormonal Test (*n* = 1243)	*p* Value
Count, mean ± SD (M/mL)	62.1 ± 45.7	54.7 ± 46.4	0.060
Motility, mean ± SD (%)	43.3 ± 17.0	40.2 ± 18.8	0.051
Normal forms, mean ± SD (%)	8.4 ± 5.0	8.0 ± 4.9	0.391
Age, mean ± SD (years)	37.6 ± 6.9	39.7 ± 8.0	0.002
Primary infertility (%)	59.0	56.5	0.612
Secondary infertility (%)	41.0	43.5	0.612
Varicocele present (%)	10.3	9.3	0.820
Smoker (%)	80.1	83.1	0.415

Comparison of clinical and biological characteristics between patients who underwent hormonal evaluation (*n* = 156) and those without hormonal evaluation (*n* = 1243). *p*-values were calculated using Student’s *t*-test for quantitative variables and the chi-square test for qualitative variables.

**Table 3:** Semen characteristics of the primary and secondary infertility groups.

Parameter	Primary Infertility (*n* = 338 (86%))	Secondary Infertility (*n* = 55 (14%))	*p* Value
Age, mean ± SD	39.0 ± 7.34	45.5 ± 8.46	<0.001
Duration, mean ± SD	4.28 ± 3.73	3.96 ± 4.37	0.6
Motility, mean ± SD	41.1 ± 18.5	41.2 ± 19.0	0.97
Count, mean ± SD	55.6 ± 48.1	67.3 ± 58.9	0.16
Normal forms, mean ± SD	8.11 ± 4.81	9.00 ± 5.44	0.26

Comparison of biological characteristics between patients with primary infertility (*n* = 338) and those with secondary infertility (*n* = 55). *p*-values were calculated using Student’s *t*-test.

**Table 4:** Characteristics of semen from varicocele carrier and non-carrier groups.

Parameter	Varicocele No (*n* = 589)	Varicocele Yes (*n* = 124)	*p* Value
Age, mean ± SD	39.5 ± 8.14	36.0 ± 8.29	<0.001
Volume, mean ± SD	3.36 ± 1.46	3.45 ± 1.55	0.54
Motility, mean ± SD	42.3 ± 17.4	37.3 ± 19.1	0.00724
Count, mean ± SD	60.0 ± 50.0	44.2 ± 43.2	<0.001
Normal forms, mean ± SD	8.26 ± 4.69	7.58 ± 4.30	0.11
Live mean, ± SD	71.0 ± 14.3	71.0 ± 14.3	0.075

Comparison of biological characteristics between patients with a history of varicocele (*n* = 124) and those without a history of varicocele (*n* = 589). *p*-values were calculated using Student’s *t*-test.

**Table 5:** Factors associated with oligozoospermia—Univariate analysis.

Variable	Crude OR	95% CI Lower	95% CI Upper	*p*-Value
Age (years)	0.998	0.983	1.013	0.766
Smoking	0.659	0.438	0.992	0.046
Varicocele	1.812	1.229	2.671	0.003
High FSH	7.252	3.149	16.704	<0.001
Low Inhibin B	Unstable result	Not interpretable	Not interpretable	Not interpretable
Low Testosterone	Non-convergent model	Not calculable	Not calculable	Not calculable

## Data Availability

Data can be requested from the corresponding author.
